# Supplementary figures and images for: rahu is a mutant allele of Dnmt3c, encoding a DNA methyltransferase homolog required for meiosis and transposon repression in the mouse male germline
Source: PLoS Genet. 2017 Aug 30;13(8):e1006964. doi: 10.1371/journal.pgen.1006964 (PMC5607212; doi:10.1371/journal.pgen.1006964)

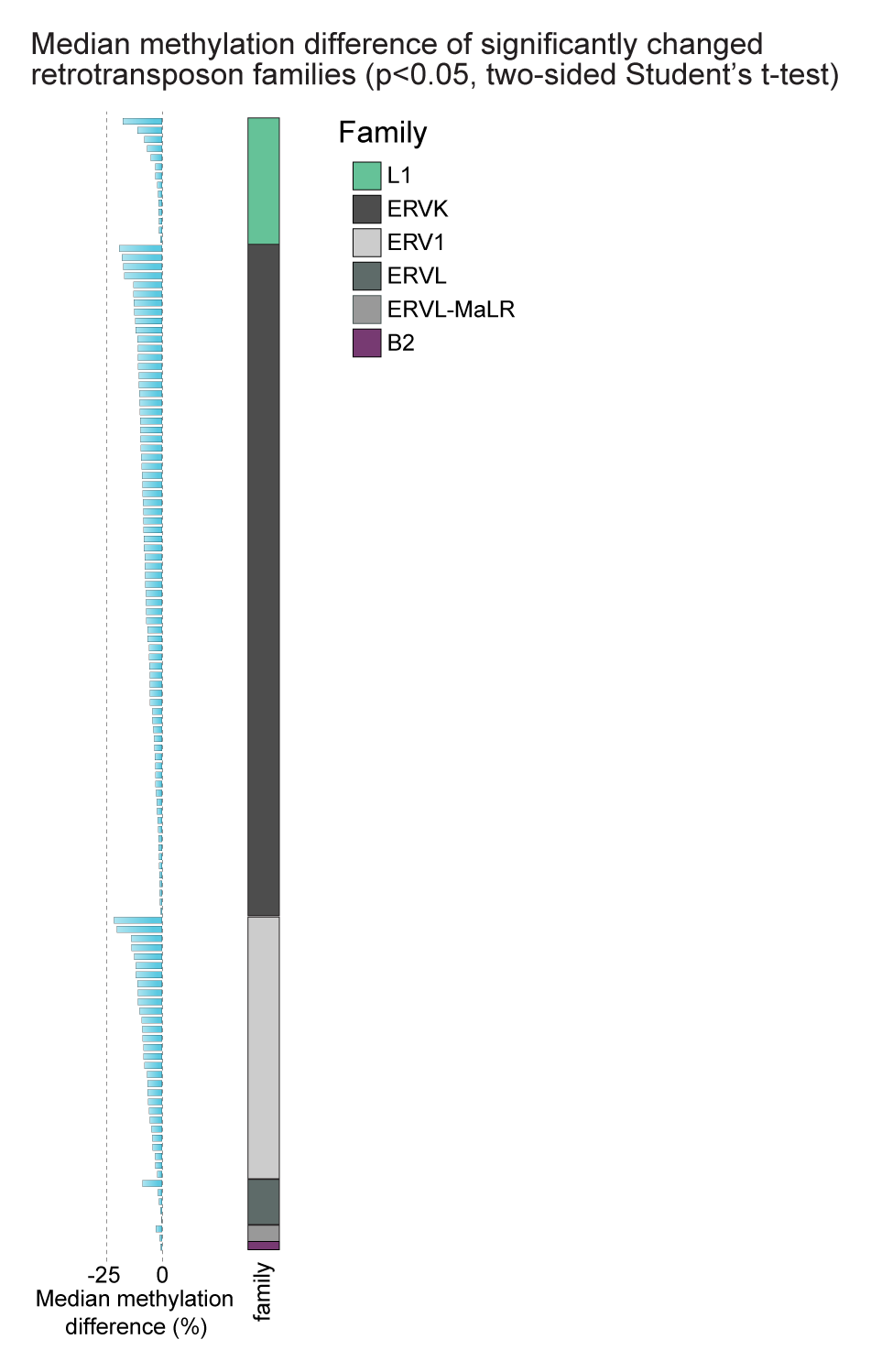

Supplement: S1 Fig — (TIF) [file pgen.1006964.s006.tif]
